# 867. Diagnostic test accuracy of TRAIL, IP-10 and C-RP biomarker combination (MeMed BV test) for discrimination of viral vs. bacterial infections among Emergency Department patients with febrile illness: A Systematic Review and Meta-analysis

**DOI:** 10.1093/ofid/ofad500.912

**Published:** 2023-11-27

**Authors:** Manasa Kandula, John J Farrell

**Affiliations:** UIC - Peoria, Peoria, Illinois; University of Illinois College of Medicine, Peoria, IL

## Abstract

**Background:**

Infections (eg. RSV, influenza & *Streptococcus pyogenes*) have surged following discontinuation of social distancing and masking. Emergency Department (ED) providers feel pressure to prescribe antibiotics for common presentations such as fever for fear of missing a case of Group A Strep, which can lead to antibiotic misuse. Multiplex PCR assays are costly and cultures take 1-2 days to result, and lack sensitivity when antibiotics have already been started. There is a need for a rapid, affordable, and accurate method to distinguish bacterial from viral infections. Some prospective studies have demonstrated that a diagnostic assay that combines CRP with IP-10 and TRAIL can effectively assist ED providers with decisions regarding antibiotic therapy in patients with a febrile illness.

**Figure:**
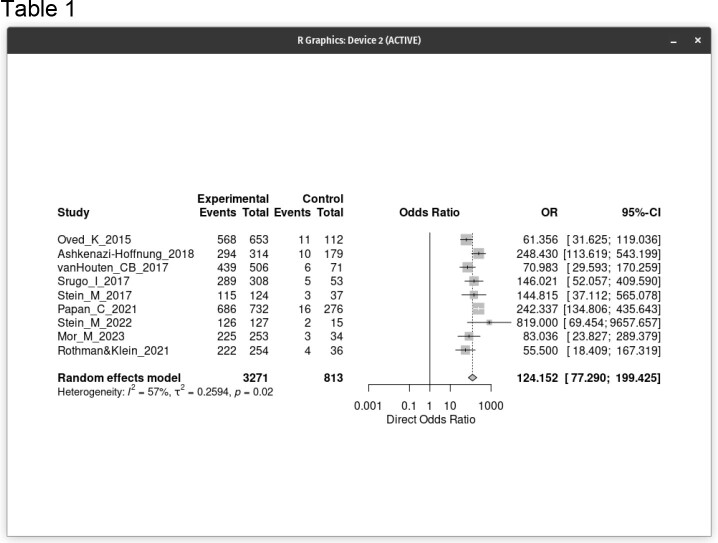

Forrest plot of the individual and composite direct odds ratios (DOT) respectively for the nine studies included in the meta-analysis

**Figure:**
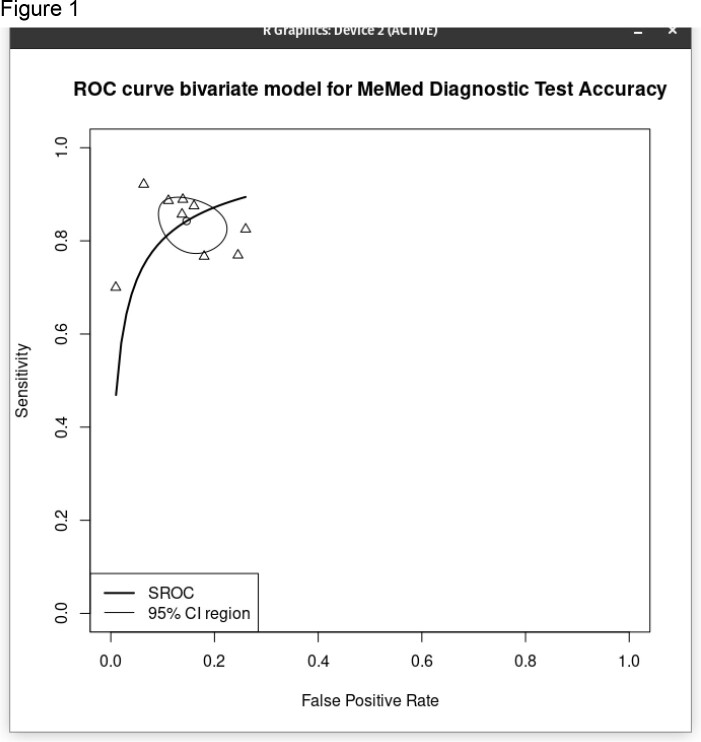

Summary Receiver Operating Characteristic (SROC) analysis of the diagnostic accuracy of the MeMed BV test. The SROC analysis was applied to the data which was pooled from all nine studies included in this meta-analysis

**Methods:**

The analysis was registered with PROSPERO prior to following PRISMA-DTA framework, which yielded 9 studies with 4X4 tables for sensitivity, specificity, and diagnostic accuracy of the BV test for bacterial vs. viral infection. Articles were searched in PubMed, Medline, Google Scholar and Ovid. Random effects models were used to calculate pooled proportions. The estimated total effect sizes, test for heterogeneity and moderator effect, and ROC curve are reported using R software.

**Results:**

9 prospective studies were included with a total of 4084 patients (3271 cases and 813 controls). The proportion of the random effect model was 0.841 (95% CI, 0.788 to 0.882). The total effect sizes of all nine studies are shown. The OR of the random effect model is 124.2 (95% CI, 77.3 to 199.4) and p-value < 0.0001, meaning the OR of a positive BV test result among persons with disease was approximately 124 times higher than the OR for a positive test among persons with no disease. The bivariate diagnostic random-effects meta-analysis for viral vs. bacterial infection AUC = 0.973.

**Conclusion:**

Across 9 studies performed over 8 years, the combination of TRAIL, IP-10 and C-RP biomarkers (BV test) accurately distinguished between bacterial and viral infections in patients with febrile illness. The test has the potential to be used to facilitate timely diagnosis and antimicrobial treatment decisions. Further large scale studies are needed to establish its role in antimicrobial stewardship.

**Disclosures:**

**All Authors**: No reported disclosures

